# Acne and risk of mental disorders: A two-sample Mendelian randomization study based on large genome-wide association data

**DOI:** 10.3389/fpubh.2023.1156522

**Published:** 2023-03-31

**Authors:** Lin Liu, Yuzhou Xue, Yangmei Chen, Tingqiao Chen, Judan Zhong, Xinyi Shao, Jin Chen

**Affiliations:** ^1^Department of Dermatology, The First Affiliated Hospital of Chongqing Medical University, Chongqing, China; ^2^Department of Cardiology and Institute of Vascular Medicine, Peking University Third Hospital, Beijing, China

**Keywords:** acne, mental disorders, Mendelian randomization, GWAS, schizophrenia

## Abstract

**Background:**

Despite a growing body of evidence that acne impacts mental disorders, the actual causality has not been established for the possible presence of recall bias and confounders in observational studies.

**Methods:**

We performed a two-sample Mendelian randomization (MR) analysis to evaluate the effect of acne on the risk of six common mental disorders, i.e., depression, anxiety, schizophrenia, obsessive–compulsive disorder (OCD), bipolar disorder, and post-traumatic stress disorder (PTSD). We acquired genetic instruments for assessing acne from the largest genome-wide association study (GWAS) of acne (*N* = 615,396) and collected summary statistics from the largest available GWAS for depression (*N* = 500,199), anxiety (*N* = 17,310), schizophrenia (*N* = 130,644), OCD (*N* = 9,725), bipolar disorder (*N* = 413,466), and PTSD (*N* = 174,659). Next, we performed the two-sample MR analysis using four methods: inverse-variance weighted method, MR-Egger, weighted median, and MR pleiotropy residual sum and outliers. Sensitivity analysis was also performed for heterogeneity and pleiotropy tests.

**Results:**

There was no evidence of a causal impact of acne on the risk of depression [odds ratio (OR): 1.002, *p* = 0.874], anxiety (OR: 0.961, *p* = 0.49), OCD (OR: 0.979, *p* = 0.741), bipolar disorder (OR: 0.972, *p* = 0.261), and PTSD (OR: 1.054, *p* = 0.069). Moreover, a mild protective effect of acne against schizophrenia was observed (OR: 0.944; *p* = 0.033).

**Conclusion:**

The increased prevalence of mental disorders observed in patients with acne in clinical practice was caused by modifiable factors, and was not a direct outcome of acne. Therefore, strategies targeting the elimination of potential factors and minimization of the occurrence of adverse mental events in acne should be implemented.

## 1. Introduction

Acne is a common facial chronic inflammatory skin disease that affects 9.4% of the global population (1). Despite being a non-threatening life condition, acne has been reported to have a psychological impact on both adolescents (2) and adults (3, 4). The most substantial acne-related mental disorders are depression and anxiety. Clinical observational studies have indicated an increased risk of depression and anxiety in patients with acne (5, 6). However, a few studies found no significant results related to the association of acne on depression (7). As for other mental disorders, in a cohort study, the prevalence of post-traumatic stress disorder (PTSD) was higher in patients with acne than in controls, and the prevalence of obsessive–compulsive disorder (OCD) was similar between patients and controls (8). However, in another cross-sectional design, the prevalence of OCD was higher in patients with acne than in controls (9). Therefore, the associations between acne and mental disorders remain unclear.

Nevertheless, considering that these clinical observational analyses were susceptible to uncontrolled confounders, such as the use of isotretinoin (10, 11), and that the association of acne with mental disorders remains controversial and causality has not been established, we attempted to find an appropriate approach to further investigate these problems. Two-sample Mendelian randomization (MR) analysis provided a method to determine the causality of exposures and outcomes using genetic variants as instrumental variables for exposure, which was not subject to the influence that vitiate conventional study designs and could minimize potential confounders (12).

In this study, based on genome-wide association studies (GWAS) data from large samples, we performed a two-sample MR study to investigate the association of acne with six common mental disorders: depression, anxiety, schizophrenia, OCD, bipolar disorder, and PTSD.

## 2. Materials and methods

This study aimed to explore the association between acne and the risk of mental disorders including depression, anxiety, schizophrenia, OCD, bipolar disorder, and PTSD. To comprehensively evaluate these relationships and to follow the principle that samples from exposure and outcomes should have as little duplication as possible, we systematically searched the PubMed dataset from inception to November 18, 2022, and acquired GWAS data of large European ancestry samples. Next, we conducted two-sample MR analyses of acne on each mental disorder based on three assumptions of MR: genetic instrumental variables are associated with exposure, i.e., relevance assumption; genetic instrumental variables are independent of confounders, i.e., independence assumption; and genetic instrumental variables are irrelevant to outcome, i.e., exclusion restriction assumption.

### 2.1. Genetic instrumental variables for acne

We obtained genetic instrumental variables for assessing acne from the most recent and largest GWAS meta-analysis of acne in nine independent European ancestry cohorts, which comprised 20,165 cases and 595,231 controls (13). The definitions of acne in these cohorts varied across clinical assessments, electronic health records, and self-report questionnaires. A total of 46 independent, genome-wide significant single-nucleotide polymorphisms (SNPs) were identified using linkage disequilibrium clumping with a *p*-value cut-off of 5 × 10^−8^, 1 Mb distance between variants, and *R*^2^ < 0.001 for variants within the genomic distance cut-off. Herein, these conditions for filtering SNPs confirmed the correlation, independence, and statistical intensity settings of SNPs.

### 2.2. Summary statistics for mental disorders

We selected six common mental disorders as outcomes: depression, anxiety, schizophrenia, OCD, bipolar disorder, and PTSD. The summary statistics for depression included 170,756 cases and 329,443 controls from 33 studies in the Psychiatric Genomics Consortium (PGC) 139 k and the United Kingdom Biobank, which was reported in the largest European GWAS meta-analysis of depression (14). Regarding anxiety, we collected data of seven European samples on anxiety from the Anxiety NeuroGenetics STudy Consortium, totaling 5,580 cases and 11,730 controls (15). The diagnosis of anxiety disorder was mostly based on the diagnostic and statistical manual of mental disorders (DSM) and involved the use of standardized assessment instruments. The summary statistics for schizophrenia were derived from the newest and largest GWAS meta-analysis based on 90 cohorts in the PGC dataset (16). Considering that we concentrated on European ancestry, we extracted the European samples only. Thus, data from 76 cohorts including 53,386 cases and 77,258 controls were included in our analysis. The summary statistics for OCD were obtained from a study of seven subsamples, investigating a total of 2,688 individuals of European ancestry with OCD and 7,037 matched controls (10). All individuals met the DSM-IV criteria for OCD. For bipolar disorder, we collected data from a GWAS meta-analysis of 57 cohorts in Europe, North America, and Australia, which contained a total of 41,917 cases and 371,549 controls (17). The cases were diagnosed by international consensus criteria (DSM-IV, ICD-9, or ICD-10) for lifetime bipolar disorder based on structured diagnostic interviews, clinician-administered checklists, and medical record reviews. The summary statistics of PTSD were obtained from the PGC-PTSD Freeze 2, comprising an ancestrally diverse group of 23,212 cases and 151,447 controls from 60 different PTSD studies. The diagnostic criteria were self-reported PTSD or presence of clinical features.

### 2.3. Primary analysis

Considering the MR assumptions, we excluded SNPs that had a correlation with outcomes (*p* < 5 × 10^−8^) in the summary statistics. We then used data harmonization to ensure a consistent direction of SNP alleles in the exposure and outcomes. We adopted four methods to perform MR analysis: inverse-variance weighted (IVW) method, MR-Egger, weighted median, and MR pleiotropy residual sum and outlier (MR PRESSO). The random effects model of the IVW method was considered the main method for determining the associations between exposure and outcomes. The other three methods were conducted to supplement the IVW results to completely measure the associations between acne and mental disorders from multiple perspectives. If there were potentially pleiotropic SNPs, called “outliers,” MR PRESSO could provide corrected results by removing them. Forest plots and scatter plots were used to visualize the primary analysis results.

### 2.4. Sensitivity analysis

For heterogeneity analysis, we used Cochran’s Q statistic for IVW and MR-Egger (*p*-value cut-off = 0.05). A funnel plot was used to visualize heterogeneity. Leave-one-out analysis was performed to evaluate the influence of each SNP on the results by eliminating one SNP at a time. In addition, horizontal pleiotropy was checked using the MR-Egger intercept (*p*-value cut-off = 0.05). The MR PRESSO global test was also used as a supplement to detect the presence of horizontal pleiotropy. If there were outliers in the MR PRESSO test, the MR PRESSO distortion test examined whether there were differences in the results before and after the removal of outliers.

All statistical analyses were performed with R software 4.1.0 using the ‘TwoSampleMR’ package and ‘MR PRESSO’ package.

## 3. Results

### 3.1. Mendelian randomization analysis of acne on mental disorders

The SNPs used as instrumental genetic variables for acne are listed in Supplementary Table 1. Among these mental disorders, MR analysis of IVW did not reveal that acne could increase the risk of mental disorders. There were no effects of genetically instrumented acne on depression [odds ratio (OR): 1.002, 95% confidence interval (CI): 0.982–1.022; *p* = 0.874], anxiety (OR: 0.961, 95% CI: 0.848–1.074; *p* = 0.49), OCD (OR: 0.979, 95% CI: 0.856–1.103; *p* = 0.741), bipolar disorder (OR: 0.972, 95% CI: 0.922–1.022; *p* = 0.261), and PTSD (OR: 1.054, 95% CI: 0.997–1.111; *p* = 0.069).

**Figure 1:**
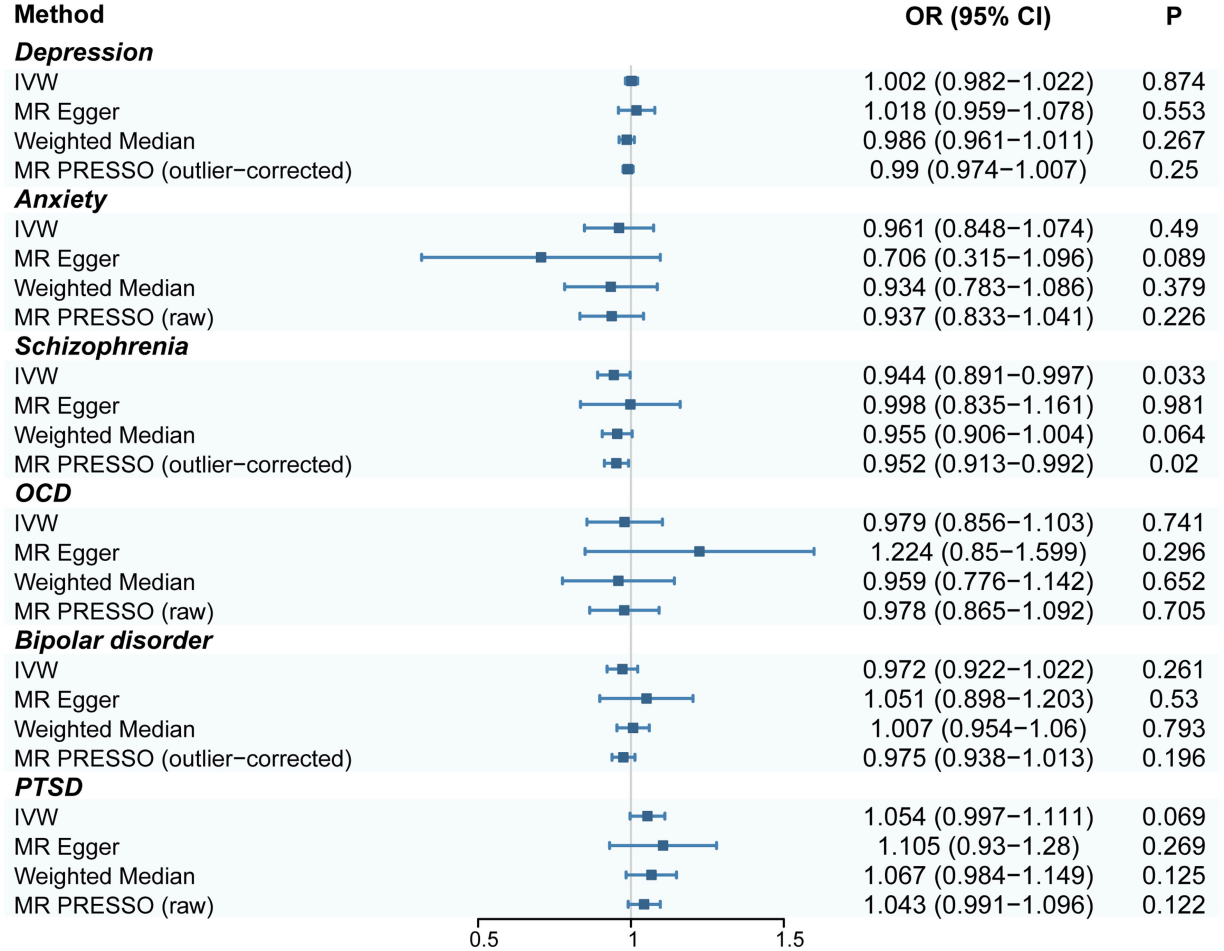
Forest plot of estimates for the association of acne on depression, anxiety, schizophrenia, OCD, bipolar disorder, and PTSD. CI, confidence interval; OR, odds ratio. IVW, inverse-variance weighted; MR PRESSO, MR pleiotropy residual sum and outlier; OCD, obsessive–compulsive disorder; PTSD, post-traumatic stress disorder.

However, our results showed that genetic liability to acne mildly decreased the risk of schizophrenia (OR: 0.944, 95% CI: 0.891–0.997; *p* = 0.033). The results of the MR PRESSO method, even after removing outliers, showed the same result for acne in patients with schizophrenia (OR: 0.952, 95% CI: 0.913–0.992; *p* = 0.02). The results of all methods are shown in Figure 1 and Supplementary Figure 1.

### 3.2. Sensitivity analysis

The sensitivity analysis results were generally consistent with the main results. Heterogeneity was observed only in depression, schizophrenia, and bipolar disorder (Cochran’s *Q p* < 0.05). The other mental disorders, including anxiety, OCD, and PTSD, were not heterogeneous, according to Cochran’s *Q* test. Visual evaluation of funnel plots revealed no significant bias (Supplementary Figure 2). The leave-one-out analysis suggested that each genetic instrumental variable for acne included in the analysis remained generally consistent in their impact on the outcomes (Supplementary Figure 3).

Regarding pleiotropy, the MR-Egger intercept test did not indicate directional pleiotropy in any analysis. The MR PRESSO global test indicated the presence of pleiotropic effects of depression, schizophrenia, and bipolar disorder (*p* = 0.013, < 0.001, and < 0.001, respectively). However, after removing outliers, the MR PRESSO distortion test did not show any significant results (*p* = 0.754, 0.114, and 0.436, respectively). The results of sensitivity analysis are listed in Table 1.

**Table 1:** Results of primary and sensitivity analysis of acne on mental disorders.

Method	Beta	SE	*p*-Value	Heterogeneity test		Pleiotropy test		
				Cochran’s Q	Q_P-value	P-Intercep*t* test	P-Global test	P-Distortion test
*Depression*								
IVW	0.002	0.010	0.874	58.409	0.014			
MR Egger	0.018	0.031	0.553	57.868	0.012	0.565		
Weighted median	−0.014	0.013	0.267					
MR PRESSO Raw	−0.006	0.009	0.487					
MR PRESSO Outlier-corrected	−0.010	0.008	0.250				0.013	0.754
*Anxiety*								
IVW	−0.040	0.058	0.490	41.906	0.266			
MR Egger	−0.349	0.199	0.089	39.076	0.333	0.115		
Weighted median	−0.068	0.077	0.379					
MR PRESSO Raw	−0.065	0.053	0.226				0.227	
*Schizophrenia*								
IVW	−0.057	0.027	0.033	95.821	0.000			
MR Egger	−0.002	0.083	0.981	94.480	0.000	0.486		
Weighted median	−0.046	0.025	0.064					
MR PRESSO Raw	−0.069	0.024	0.007					
MR PRESSO Outlier-corrected	−0.049	0.020	0.020				<0.001	0.114
*OCD*								
IVW	−0.021	0.063	0.741	33.616	0.672			
MR Egger	0.203	0.191	0.296	32.085	0.699	0.224		
Weighted median	−0.042	0.093	0.652					
MR PRESSO Raw	−0.022	0.058	0.705				0.473	
*Bipolar disorder*								
IVW	−0.028	0.025	0.261	74.952	0.000			
MR Egger	0.049	0.078	0.530	72.698	0.000	0.298		
Weighted median	0.007	0.027	0.793					
MR PRESSO Raw	−0.034	0.021	0.123					
MR PRESSO Outlier-corrected	−0.025	0.019	0.196				<0.001	0.436
*PTSD*								
IVW	0.053	0.029	0.069	41.970	0.303			
MR Egger	0.100	0.089	0.269	41.617	0.277	0.579		
Weighted median	0.064	0.042	0.125					
MR PRESSO Raw	0.042	0.027	0.122				0.329	

IVW, inverse variance weighted; MR PRESSO, MR pleiotropy residual sum and outlier; OCD, obsessive compulsive disorder; PTSD, post-traumatic stress disorder.

## 4. Discussion

To the best of our knowledge, this is the first study to perform MR analysis for investigating the association between acne and the risk of mental disorders. To improve reliability, we collected the largest GWAS data on acne and mental disorders to the extent available. The included data were from large cohorts of European ancestry. MR analyses revealed that genetically predicted acne was unrelated to depression, anxiety, OCD, bipolar disorder, and PTSD. In addition, it might decrease the risk of schizophrenia development.

This finding is inconsistent with the findings of a previous observational study, which reported an increased risk of mental disorders, mainly depression and anxiety, in patients with acne. A large meta-analysis of studies involving 1,029,299 participants from 35 studies on depression and 21,634 participants from 24 studies on anxiety reported significant associations of acne with depression and anxiety (*r* = 0.22 and 0.25) (5). An observational study on a Taiwanese population of 1 million individuals also suggested an additive risk of major depression in patients with acne (18). Although there are only a few related studies till date, acne has been reported to be related to OCD (9, 19), and skin picking disorder has been identified by the DSM-5 as a related key symptom (20). Additionally, patients with acne were reported to be at high risk of developing PTSD in a cross-sectional study of 410 people (21). However, these are findings of clinical observational studies; thus, recall bias and confounders might exist due to the limitation of the study design.

On the other hand, some factors, such as isotretinoin use, further confused and complicated this problem. Since the introduction of isotretinoin, many studies have reported a correlation between isotretinoin use and the risk of potential psychiatric side effects, particularly depression and suicide (22). Isotretinoin was even listed among the top 10 drugs associated with depression as the only non-psychotropic drug (23). In addition, exacerbation of bipolar disorder symptoms and a possible link with psychosis were reported in patients under treatment with isotretinoin (23). However, several studies reported that isotretinoin could not increase the risk of mental disorders, but there was no evidence, whereas some suggested that it could improve psychiatric symptoms (11, 24, 25). A prospective study showed that oral drugs for acne, including isotretinoin and antibiotics, could decrease social anxiety and improve OCD symptoms in patients with acne (26). Moreover, a recent global study showed that isotretinoin could decrease the risk of psychiatric disturbances, including depression, anxiety, bipolar disorder, PTSD, schizophrenia, and adjustment disorder, whereas an increased risk of suicidal ideation was found in patients with acne under isotretinoin treatment (10). Therefore, this association remains unclear, leaving unanswered questions: is there a causal relationship between acne and mental disorders? Is isotretinoin a confounder, protective factor, or driver?

Here, MR analysis made it possible to reveal the real causality between acne and mental disorders using genetic variants as instrumental variables and decreasing the role of confounders. In this study, no causality was found in the relationship of acne with depression, anxiety, OCD, bipolar disorder, or PTSD. This finding suggests that although previous studies have reported that acne is related to mental health burden in clinical practice (27), it seemed more likely to be related to some changeable factors rather than being directly caused by genetic liability to acne, possibly representing the sum of acne downstream results, such as side effects of drugs, unhealthy lifestyle, and preoccupation with appearance among peers. Since all these factors could be changed by implementing appropriate actions, the rate of adverse psychiatric events in patients with acne could theoretically be significantly reduced.

The significant psychosocial distress in patients with acne might come from the deteriorating appearance, young age of the sufferers, and low quality of life, particularly in resistant or persistent moderate to severe acne (28). The embarrassment about the appearance would trigger impairment of social activities and the aggravation of psychosocial anguish (29). And it has been reported that using the measurements of quality of life, such as dermatology life quality index, Cardiff acne disability index, and Turkish Acne Quality of Life Index, the scores are lower in patients with acne than healthy people (30–34). Beside, a significant association between quality of life of patients with acne and their anxiety and depression levels has been revealed (35). Furthermore, it has been reported low quality of life lead that people suffering from acne often withdraw from public life, causing social isolation and even suicidal ideation (36). Therefore, mental health management in patients with acne deserves more attention and can be organically combined with clinical management.

In this study, we found that genetic liability to acne was negatively associated with schizophrenia. There have been few previous reports on acne and schizophrenia. The only study we searched was the report by Kridin et al. (10) on a decreased risk of schizophrenia in patients treated with isotretinoin compared with those treated with antibiotics. However, isotretinoin is usually used to treat resistant or persistent moderate-to-severe acne (28), and there might be selection bias between the isotretinoin-treated group and antibiotics-treated group. More severe cases were selected, and the genetic factor of acne might play a protective role as reported in the study by Kridin et al. The underlying genetic correlation between acne and isotretinoin warrants further investigation.

Our study has some limitations. First, our study was based on a population of primarily European ancestry, and the conditions in non-European populations are unclear. Second, because of data limitations, we could not further perform a subgroup analysis of variables such as sex, age, and region. Third, our study could neither confirm whether the increased prevalence of depression and anxiety in acne was due to isotretinoin use nor confirm the actual role of isotretinoin in this problem. This requires further investigation using approaches other than MR analysis.

In conclusion, our MR study provided no evidence of a causal effect of acne on depression, anxiety, OCD, bipolar disorder, or PTSD. Furthermore, acne has been found to play a protective genetic role in the development of schizophrenia. In addition, more actions on relevant modifiable factors might help prevent mental disorders in patients with acne.

## Data availability statement

Publicly available datasets were analyzed in this study. This data can be found here: Genetic instrumental variables of acne can be accessed from original GWAS articles https://www.nature.com/articles/s41467-022-28252-5. Summary statistics of depression could be downloaded from https://datashare.ed.ac.uk/handle/10283/3203. Summary statistics of anxiety could be downloaded from https://figshare.com/articles/dataset/anx2016/14842689. Summary statistics of schizophrenia could be downloaded from https://figshare.com/articles/dataset/scz2022/19426775. Summary statistics of OCD could be downloaded from https://figshare.com/articles/dataset/ocd2018/14672103. Summary statistics of bipolar disorder could be downloaded from https://figshare.com/articles/dataset/PGC3_bipolar_disorder_GWAS_summary_statistics/14102594. Summary statistics of PTSD could be downloaded from https://figshare.com/articles/dataset/ptsd2019/14672133.

## Ethics statement

Ethical review and approval was not required for the study on human participants in accordance with the local legislation and institutional requirements. Written informed consent for participation was not required for this study in accordance with the national legislation and the institutional requirements.

## Author contributions

LL and JC: conceptualization. YX and LL: data acquisition and processing. YC and LL: Interpretation of data. YX and LL: software. LL was responsible for writing the initial article. TC, JZ, and XS: revision and finalization. All authors contributed to the article and approved the submitted version.

## Funding

This study was supported by National Natural Science Foundation of China (n82073462).

## Conflict of interest

The authors declare that the research was conducted in the absence of any commercial or financial relationships that could be construed as a potential conflict of interest.

## Publisher’s note

All claims expressed in this article are solely those of the authors and do not necessarily represent those of their affiliated organizations, or those of the publisher, the editors and the reviewers. Any product that may be evaluated in this article, or claim that may be made by its manufacturer, is not guaranteed or endorsed by the publisher.
