# Supplementary material for: Acne and risk of mental disorders: A two-sample Mendelian randomization study based on large genome-wide association data
Source: Front Public Health. 2023 Mar 31;11:1156522. doi: 10.3389/fpubh.2023.1156522 (PMC10102334; doi:10.3389/fpubh.2023.1156522)
Supplement: Supplementary file 1 [file Data_Sheet_1.docx]

Supplementary Material

# Supplementary Tables

**Supplementary Table 1**. SNPs used as genetic instrumental variables for acne

# Supplementary Figures


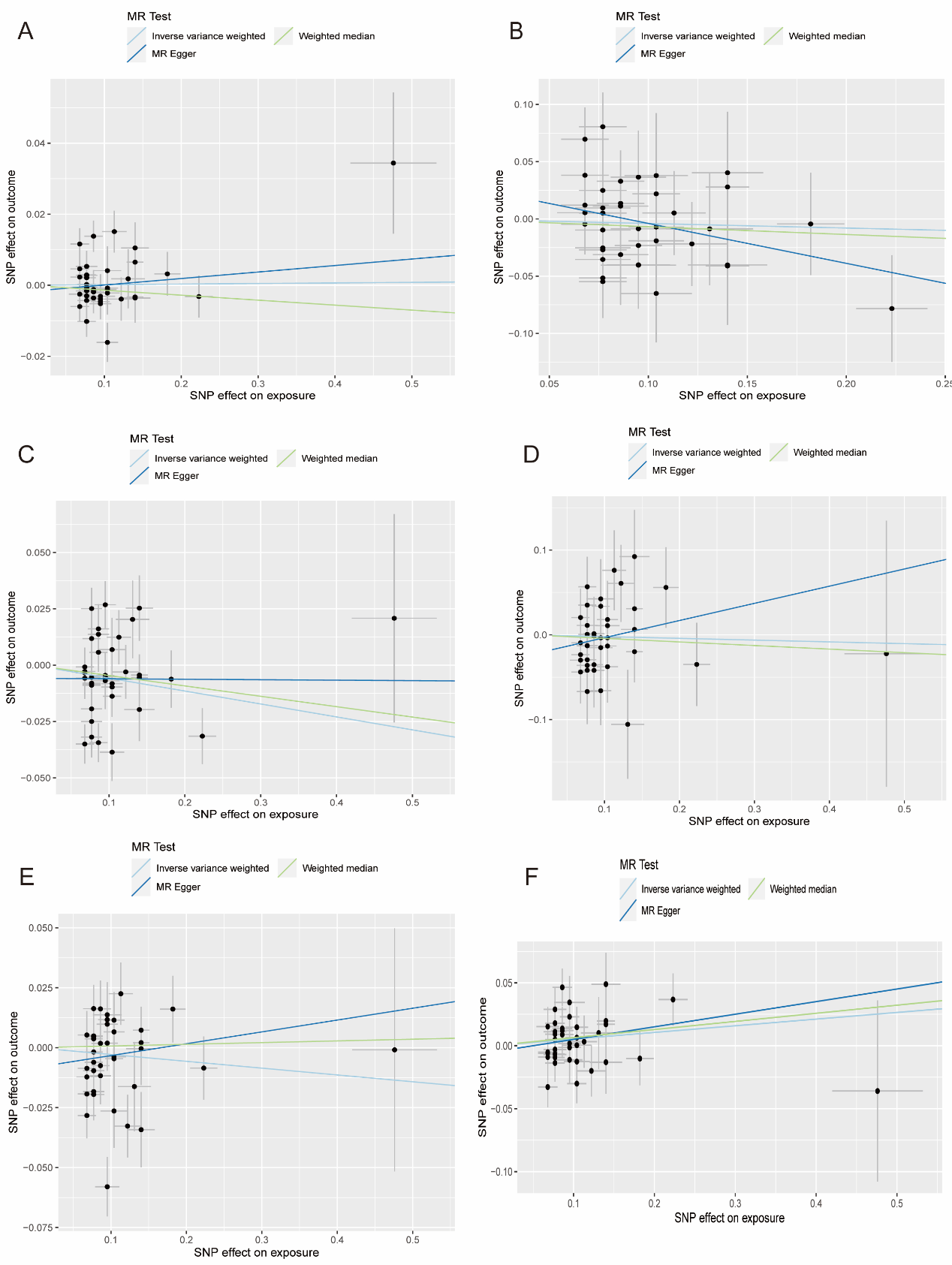


**Supplementary Figure 1**. Scatter plots of estimates for the association of acne on (A) depression, (B) anxiety, (C) schizophrenia, (D) obsessive-compulsive disorder, (E) bipolar disorder, and (F) post-traumatic stress disorder.


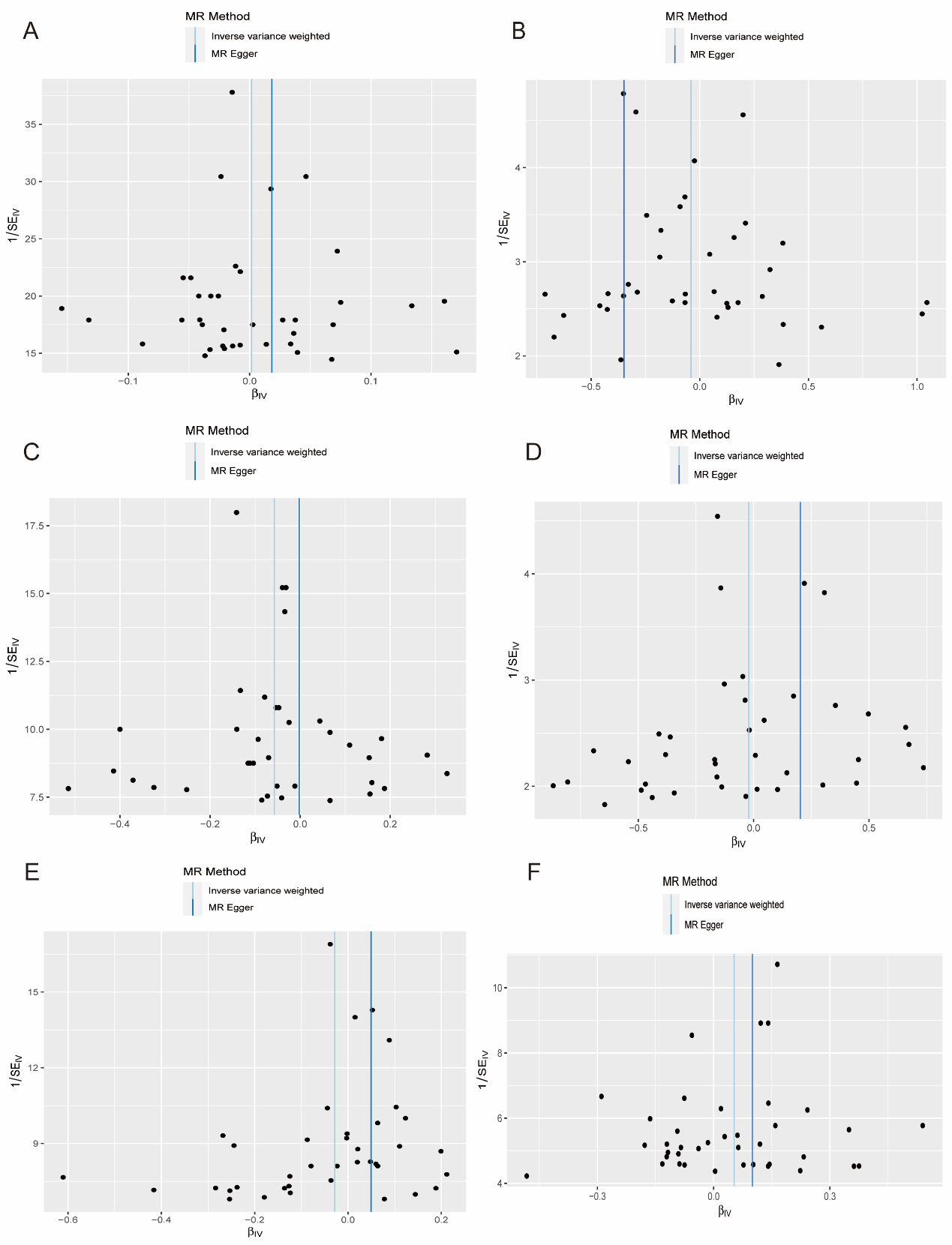


**Supplementary Figure 2**. Funnel plots of estimates for the association of acne on (A) depression, (B) anxiety, (C) schizophrenia, (D) obsessive-compulsive disorder, (E) bipolar disorder, and (F) post-traumatic stress disorder.


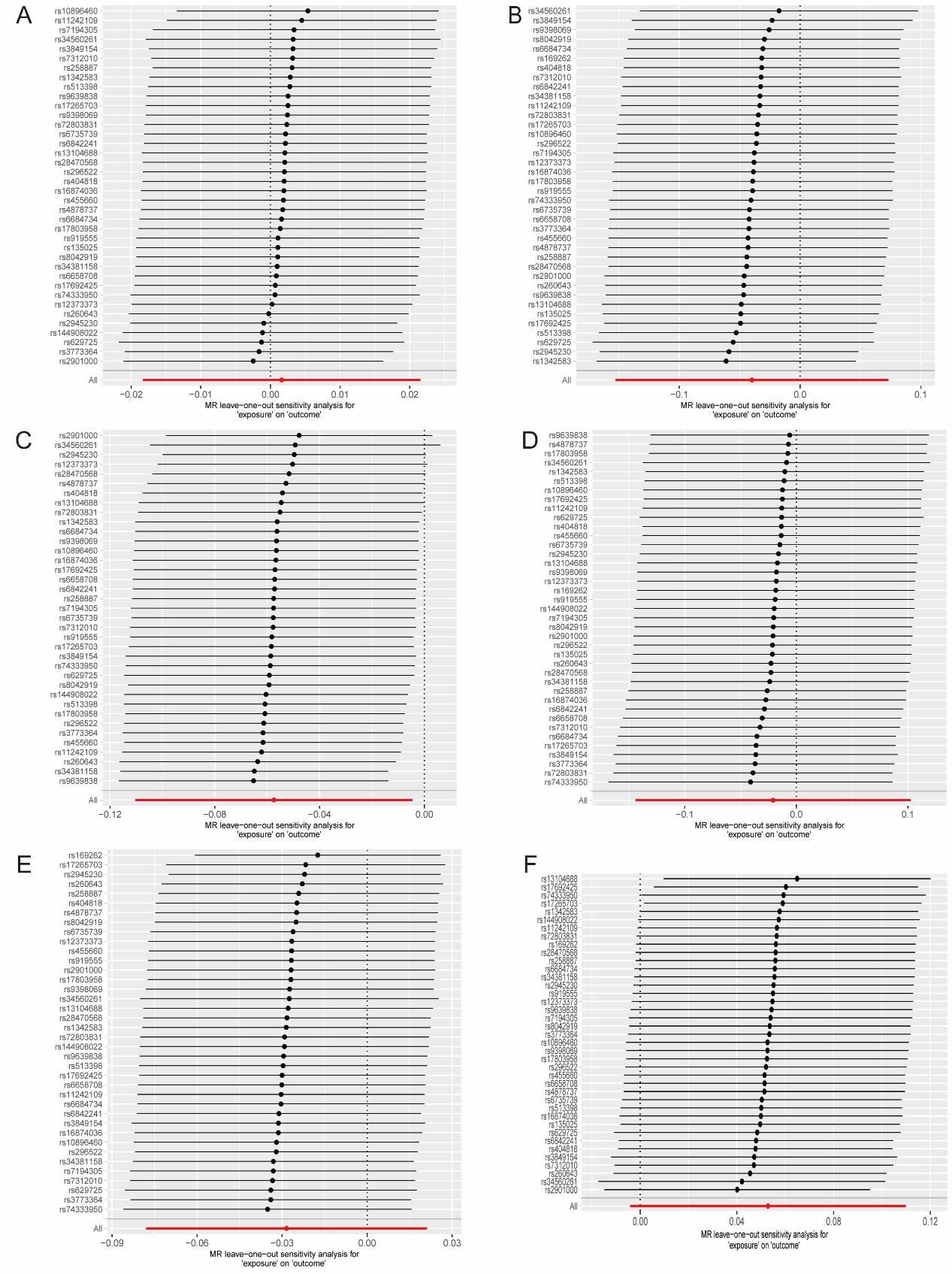


**Supplementary Figure 3**. Leave-one-out results for acne on (A) depression, (B) anxiety, (C) schizophrenia, (D) obsessive-compulsive disorder, (E) bipolar disorder, and (F) post-traumatic stress disorder.
