# Supplementary material for: Acne and risk of mental disorders: A two-sample Mendelian randomization study based on large genome-wide association data
Source: Front Public Health. 2023 Mar 31;11:1156522. doi: 10.3389/fpubh.2023.1156522 (PMC10102334; doi:10.3389/fpubh.2023.1156522)
Supplement: Supplementary file 2 [file Table_1.docx]

**Supplementary Table 1. SNPs used as genetic instrumental variables for acne.**

| SNP | CHR | Risk allele frequency | Effect_allele | Other_allele | OR (95% CI) | Beta | SE | P-value | Putative causal gene |
| --- | --- | --- | --- | --- | --- | --- | --- | --- | --- |
| rs9639838 | 7 | 0.2087 | T | C | 1.1 (1.07–1.13) | 0.095 | 0.014 | 3.27E-11 | SUGCT |
| rs9398069 | 6 | 0.6127 | T | C | 1.08 (1.05–1.1) | 0.077 | 0.012 | 1.29E-09 | PRDM1 |
| rs919555 | 8 | 0.5352 | C | A | 1.07 (1.04–1.09) | 0.068 | 0.012 | 1.73E-08 | SOX7 |
| rs8042919 | 15 | 0.1109 | A | G | 1.11 (1.07–1.15) | 0.104 | 0.018 | 4.48E-08 | SPPL2A*, USP50, TRPM7* |
| rs80293268 | 1 | 0.0504 | C | G | 1.21 (1.14–1.28) | 0.191 | 0.03 | 3.65E-10 | ERRFI1 |
| rs74333950 | 2 | 0.8603 | T | G | 1.2 (1.16–1.24) | 0.182 | 0.017 | 3.03E-23 | WNT10A |
| rs738409 | 22 | 0.7782 | C | G | 1.09 (1.06–1.12) | 0.086 | 0.014 | 0.00000001 | PNPLA3 |
| rs7312010 | 12 | 0.4064 | A | G | 1.1 (1.07–1.12) | 0.095 | 0.012 | 9.85E-15 | BORCS5 |
| rs72803831 | 16 | 0.8875 | G | A | 1.15 (1.1–1.19) | 0.14 | 0.02 | 2.4E-11 | ADAMTS18 |
| rs7194305 | 16 | 0.584 | A | G | 1.1 (1.07–1.13) | 0.095 | 0.014 | 2.18E-15 | CLEC16A |
| rs6842241 | 4 | 0.8622 | C | A | 1.1 (1.06–1.14) | 0.095 | 0.019 | 2.22E-08 | EDNRA |
| rs6735739 | 2 | 0.334 | T | C | 1.07 (1.04–1.1) | 0.068 | 0.014 | 4.39E-08 | IL36RN*, IL1B* |
| rs6684734 | 1 | 0.3993 | G | A | 1.08 (1.05–1.1) | 0.077 | 0.012 | 5.06E-10 | * |
| rs6658708 | 1 | 0.527 | T | G | 1.08 (1.06–1.11) | 0.077 | 0.012 | 6.19E-12 | LGR6*, PPP1R12B* |
| rs629725 | 5 | 0.3393 | T | C | 1.15 (1.12–1.17) | 0.14 | 0.011 | 3.5E-28 | FST |
| rs61744384 | 11 | 0.5586 | T | A | 1.12 (1.1–1.15) | 0.113 | 0.011 | 8.58E-22 | MAP3K11*, OVOL1, PCNX3* |
| rs513398 | 1 | 0.56 | A | G | 1.09 (1.06–1.11) | 0.086 | 0.012 | 3.69E-12 | LAMC2*, LAMC1* |
| rs4878737 | 9 | 0.2568 | T | G | 1.08 (1.05–1.11) | 0.077 | 0.014 | 0.000000014 | SHB |
| rs455660 | 5 | 0.8215 | C | T | 1.09 (1.06–1.12) | 0.086 | 0.014 | 1.42E-08 | * |
| rs404818 | 1 | 0.2531 | T | C | 1.08 (1.05–1.11) | 0.077 | 0.014 | 9.76E-09 | SOAT1 |
| rs3849154 | 11 | 0.3237 | T | G | 1.15 (1.12–1.17) | 0.14 | 0.011 | 2.22E-28 | RASSF10 |
| rs3773364 | 3 | 0.1574 | G | A | 1.12 (1.08–1.15) | 0.113 | 0.016 | 1.27E-11 | TIMP4 |
| rs34560261 | 15 | 0.8345 | C | T | 1.25 (1.21–1.3) | 0.223 | 0.018 | 2.51E-35 | SEMA4B |
| rs34381158 | 3 | 0.326 | G | A | 1.08 (1.05–1.11) | 0.077 | 0.014 | 1.67E-09 | DLG1 |
| rs296522 | 1 | 0.8192 | C | T | 1.09 (1.06–1.12) | 0.086 | 0.014 | 0.000000031 | INAVA (C1orf106) |
| rs2945230 | 8 | 0.5252 | G | A | 1.07 (1.04–1.09) | 0.068 | 0.012 | 4.61E-08 | PRAG1 |
| rs2901000 | 2 | 0.4285 | A | G | 1.09 (1.07–1.12) | 0.086 | 0.012 | 3.13E-14 | BCL11A |
| rs28470568 | 22 | 0.1448 | T | G | 1.11 (1.08–1.15) | 0.104 | 0.016 | 1.19E-09 | CRELD2*, ALG12, PIM3* |
| rs260643 | 2 | 0.0998 | A | G | 1.15 (1.11–1.19) | 0.14 | 0.018 | 2.93E-12 | EDAR |
| rs258887 | 5 | 0.5439 | A | C | 1.07 (1.04–1.09) | 0.068 | 0.012 | 2.78E-08 | FCHO2 |
| rs2070475 | 22 | 0.1641 | T | A | 1.11 (1.07–1.15) | 0.104 | 0.018 | 5.4E-10 | SPECC1L*, UPB1* |
| rs1838055 | 11 | 0.1135 | C | G | 1.11 (1.07–1.15) | 0.104 | 0.018 | 1.13E-08 | DBX1 |
| rs17803958 | 8 | 0.07166 | T | C | 1.14 (1.09–1.19) | 0.131 | 0.022 | 1.13E-08 | C8orf48 |
| rs17692425 | 6 | 0.3443 | C | T | 1.07 (1.05–1.1) | 0.068 | 0.012 | 4.16E-08 | TBX18 |
| rs17265703 | 3 | 0.1521 | G | A | 1.13 (1.09–1.17) | 0.122 | 0.018 | 1.57E-13 | CSTA |
| rs169262 | 6 | 0.8091 | C | T | 1.1 (1.07–1.14) | 0.095 | 0.016 | 3.24E-10 |  |
| rs16874036 | 5 | 0.6926 | G | A | 1.11 (1.08–1.14) | 0.104 | 0.014 | 1.22E-15 | FGF10 |
| rs158343 | 5 | 0.2006 | G | C | 1.13 (1.1–1.17) | 0.122 | 0.016 | 2.5E-16 | ANKRD55 |
| rs144908022 | 11 | 0.01025 | G | A | 1.61 (1.44–1.79) | 0.476 | 0.056 | 2.19E-18 | * |
| rs135025 | 22 | 0.4414 | A | G | 1.08 (1.05–1.1) | 0.077 | 0.012 | 1.42E-10 | TIMP3 |
| rs1342583 | 13 | 0.5695 | A | G | 1.08 (1.06–1.11) | 0.077 | 0.012 | 4.85E-11 | SPRY2 |
| rs13104688 | 4 | 0.3426 | G | T | 1.11 (1.08–1.13) | 0.104 | 0.012 | 6.28E-16 | FGF2*, SPRY1* |
| rs1256580 | 1 | 0.1517 | C | G | 1.19 (1.15–1.23) | 0.174 | 0.017 | 1.49E-27 | TGFB2*, LYPLAL1* |
| rs12373373 | 18 | 0.3977 | T | C | 1.08 (1.05–1.11) | 0.077 | 0.014 | 3.49E-08 | PARD6G |
| rs11242109 | 5 | 0.4719 | T | G | 1.08 (1.05–1.1) | 0.077 | 0.012 | 2.92E-10 | SLC22A5*, PDLIM4, SLC22A4* |
| rs10896460 | 11 | 0.8125 | T | G | 1.11 (1.08–1.14) | 0.104 | 0.014 | 2.8E-11 | MYEOV |

*OR odds ratio, CI confidence intervals*
